# Telemedicine in Sports under Extreme Conditions: Data Transmission, Remote Medical Consultations, and Diagnostic Imaging

**DOI:** 10.3390/ijerph20146371

**Published:** 2023-07-15

**Authors:** Nicola Pegoraro, Benedetta Rossini, Melchiore Giganti, Eric Brymer, Erik Monasterio, Pierre Bouchat, Francesco Feletti

**Affiliations:** 1 Dipartimento di Medicina Traslazionale e per la Romagna, Università degli Studi di Ferrara, 44122 Ferrara, Italy; 2 Humans Sciences, Faculty of Health, Southern Cross University, Southern Cross Drive, Bilinga, QLD 4225, Australia; 3 Christchurch School of Medicine, University of Otago, Hillmorton Hospital, Private Bag 4733, Christchurch 8024, New Zealand; 4 Psychological Sciences Research Institute, Université Catholique de Louvain, B-1348 Louvain-la-Neuve, Belgium; 5 Dipartimento Diagnostica per Immagini—Ausl Romagna, U.O. Radiologia—Ospedale S. Maria delle Croci, 48121 Ravenna, Italy

**Keywords:** climbing, sonography, diving, windsurfing, skiing, running, diagnostic imaging

## Abstract

Telemedical technologies provide significant benefits in sports for performance monitoring and early recognition of many medical issues, especially when sports are practised outside a regulated playing field, where participants are exposed to rapidly changing environmental conditions or specialised medical assistance is unavailable. We provide a review of the medical literature on the use of telemedicine in adventure and extreme sports. Out of 2715 unique sport citations from 4 scientific databases 16 papers met the criteria, which included all research papers exploring the use of telemedicine for monitoring performance and health status in extreme environments. Their quality was assessed by a double-anonymised review with a specifically designed four-item scoring system. Telemedicine was used in high-mountain sports (37.5%; n = 6), winter sports (18.7%; n = 3), water sports (25%; n = 4), and long-distance land sports (18.7%; n = 3). Telemedicine was used for data transfer, teleconsulting, and the execution of remote-controlled procedures, including imaging diagnostics. Telemedical technologies were also used to diagnose and treat sport-related and environmentally impacted injuries, including emergencies in three extreme conditions: high mountains, ultraendurance activities, and in/under the water. By highlighting sport-specific movement patterns or physiological and pathological responses in extreme climatic conditions and environments, telemedicine may result in better preparation and development of strategies for an in-depth understanding of the stress of the metabolic, cardiorespiratory, biomechanical, or neuromuscular system, potentially resulting in performance improvement and injury prevention.

## 1. Introduction

The evolution of communication and digital technologies is radically changing medical practice. The World Health Organisation (WHO) defines telemedicine as “The delivery of health care services, where distance is a critical factor, by all health care professionals using information and communication technologies for the exchange of valid information for diagnosis, treatment and prevention of disease and injuries, research and evaluation, and for the continuing education of health care providers, all in the interests of advancing the health of individuals and their communities” [1]. Telemedicine allows remote medical assessments and consultations [2], including remote diagnostic investigations using remote guidance and data transmission. Alongside the expansion of telemedicine technologies for health service provision, many international sports authorities have also explored its potential to assist with safer sports practice. For example, in 2018, World Sailing organised a workshop on medical support for offshore yacht racing on standards for the availability of telemedicine services (TMAS) for the various categories of offshore yacht racing [3]. In addition, live-transmitting technology was among the developments proposed by the adverse “Weather impact expert Working Group”, proactively created by the “International Olympic Committee” (IOC) to help protect the health of athletes competing in the extreme environmental conditions expected at the Tokyo Olympics 2020 [4].

Precise and reliable assessment of biomedical and biomechanical parameters in the “field” can provide considerable benefits for diagnostic assessment and treatment in remote locations far from medical assistance. For instance, in high-mountain sports such as climbing and mountaineering, remote monitoring of vital signs and data transfer have been adopted since the 1960s. Moreover, recent advances in wearable technology have increased the possibility of monitoring various parameters in the exercising athlete. Specifically, extreme sports increasingly use wearable sensors to monitor athlete and equipment performance and athlete–equipment interaction. Some noteworthy examples include underfoot force and pressure sensors and IMUs for athletic gestures’ analysis and injury risk warning systems applied in BMX, downhill mountain biking, wakeboarding, and snowboarding [5,6,7].

The present review reports experiences in telemedicine applied to adventure and extreme sports. Extreme sports have been defined as high-risk sports, where severe injury or death is a possibility and integral to the sport or activity. In contrast to this definition, the injury rate in some adventure and extreme sports is not necessarily higher than in more traditional sports. For example, despite different denominators of the available data making a comparison difficult, it is 0.2/1000 person-hours in rock climbing, 0.005–0.013/person-hours in mountaineering, 1–2/1000 days in alpine skiing, 1/1000 days in windsurfing, 0.007–0.08/pilots/year in hang-gliding, and 0.48–1.7/1000 jumps in skydiving [8,9,10,11,12]. However, it is also recognised that minor injuries are likely to be under-reported in these sports [12]. However, a low injury rate in extreme sports does not exclude the possibility of incurring life-threatening events. On the other hand, in some adventure sports, the injury rate is much higher: 16.8/1000 h of practice in downhill mountain biking, 8.4/1000 h in snowkiting, 12/1000 h in wakeboarding, and 2/1000 jumps in BASE jumping [13,14,15,16,17,18]. Finally, some extreme sports are practised in hostile environments characterised by extreme, sometimes unpredictable, environmental-related conditions, such as strong winds, humidity, temperature, and pressure, which can play a primary role in the pathogenesis of sports-related injuries and illnesses. For example, wind gusts were found to be the second leading cause of injury in snowkiting. In extreme flying sports, the percentage of incidents resulting in fatalities and severe injuries was higher with high wind speed [9,13]. Despite a low injury rate in mountaineering, physiological changes related to high altitude can confer significant risk. They may result in altitude illness (21.3% above 2500 m [8]), such as acute mountain sickness, high-altitude pulmonary oedema, and high-altitude cerebral oedema. Finally, a relevant aspect is that many extreme sports are practised in austere environments, defined as areas characterised by significant environmental hazards (e.g., heat, cold, and altitude), which can exacerbate existing medical conditions [19]. For the present review, we embraced a comprehensive definition of adventure and extreme sports based on the categories proposed by Cohen et al. [20]. Therefore, we include competitive and self-evaluative activities in which the participant is asked to perform acrobatic stunts or ultraendurance efforts or is subjected to environment-related variables. We address telemedicine in mountain sports (e.g., skiing, climbing, and mountaineering), water sports (e.g., swimming, diving), sky sports (e.g., skydiving and paragliding), ultraendurance sports (e.g., long-distance triathlon and long-distance running), and acrobatic sports (skateboarding and inline skating).

Until recently, telemedicine in extreme sports had been limited to pioneering experiences and pilot studies, based on equipment built for the purpose or adapted to allow the operation and transmission of data from austere and adverse environments. Mostly the transmitted data was simple, such as single electrocardiographic lead signals to monitor heart rate. Nowadays, integrating smart clothing with telemedicine can be used for real-time biofeedback, to provide input on improving movement and reducing human error. By gathering data from previous exercise patterns, the systems can lead to the innovation of safer movement patterns, and built-in real-time alerts can help reduce injuries during participation. Telemedicine in the “field” (for example, in high-altitude expeditions, rural environments, or offshore sailing [21,22,23]) can also provide data to assess sports-specific performance in extreme sports [24]. These may promote sports-specific skill development, guide the development of sport-specific athletic preparation, or serve as feedback for training or rehabilitation [25,26,27].

With smart clothing, telemedicine enables data acquisition and provides critical information for injury prevention and medical support without hampering athletic performance, even in acrobatic sports [28]. Finally, any solution pioneered in extreme environments has the potential to be translated to the broader population in the future. The technology and know-how for collecting and delivering medical data also has the potential for public health applications in ordinary conditions.

Therefore, the present review aims to promote awareness of and research on telemedicine in adventure and extreme sports.

Considering the specific use of telemedicine in this context, for this review, we define telemedicine as “the use of electronic information and communications technologies to provide and support health care when distance separates the participants” [29].

The first objective of this publication is to review is to review the scientific literature on telemedicine applied to extreme sports: the sports where telemedicine has been used, the type of telemedical activity (e.g., consultation or data transmission), the technological solutions adopted, and the parameters evaluated. The second objective is to extrapolate the data relating to the accuracy and reliability of the data produced by each telemedicine system.

## 2. Method

A review of medical literature was performed using the standardised guidelines for systematic reviews and meta-analyses proposed by Harris et al. [30] and the guidelines for meta-analysis of observational studies in epidemiology (MOOSE) [31]. We also adopted the checklist for the Preferred Reporting Items for Systematic Reviews and Meta-Analyses 2015 (PRISMA) [32]. A protocol addressing the precise identification of Participants, Interventions, Comparisons/Controls, Outcomes, and Study Design (PICOS criteria) was preliminarily registered to the International Prospective Register of Systematic Reviews (PROSPERO) [33] with the ID number CRD42020204513. Two researchers (FF and BR) searched the MEDLINE PubMed, Scopus, Web of Science, and Cochrane electronic databases. The search covered all the available literature up to March 2020; we did not specify any starting date.

We adopted a Population Intervention Comparator Outcome and Study Design (PICOS) strategy based on two comprehensive search themes to build the search criteria for electronic databases. In particular, Theme 1 included the terms: “Tele*”, “Telemedicine”, “Teleradiology”, “Telerehabilitation”, “Tele * AND Rehabilitation”, “Telepathology”, “Telenursing”, “Remote consultation”, “Emergency medical service communication systems”, and “Internet-based intervention”. In Theme 2, we included “Sports” and “Sports medicine”. We combined these two themes using the Boolean operator “and”, and terms were searched as text, abstract, or title words (an example is reported in the Supplementary Material). Each item was exploded as a MESH term and referred to its presence in the title or abstract. No additional filters or limitations were imposed during the search.

Duplicate references were preliminarily removed using manual methods. Two researchers (BR and FF) independently screened the titles and abstracts to determine the initial eligibility based on the application of the inclusion criteria reported in Table 1. Specifically, at least one of the criteria reported in the first column of the table and both reported in the second and third columns were to be satisfied. At the same time, the papers were excluded if one of the exclusion criteria was met. Blinding of researchers was adopted to reduce bias during this process. The articles for which there was indecision about eligibility were also full-text reviewed. Disagreement in eligibility decisions was resolved by consensus. Inter-rater agreement was measured with the κ statistic [34]. Finally, the authors reviewed the full text for inclusion based on the eligibility criteria. Full-text articles were retained if they met the inclusion criteria of the study design. One author (NP) completed data extraction using a standardised form, pilot-tested on ten randomly selected studies and refined accordingly. The other author (FF) merged the data, and any discrepancies in the extracted data were resolved through discussion. Since the definition of telemedicine may have changed over the years, we chose not to adopt a rigid definition for this study.

Due to the heterogeneity of the selected papers, a specifically designed four-item telemedicine-focused scoring system was set up to evaluate the studies qualitatively. The score was based on broad criteria (See Table 2) and exclusively on the quality of the exposure data relating to telemedicine. For each study, the representativeness of the cohort of sports participants studied was also assessed. Specifically, the population was considered more or less adequate, based on whether it was homogeneously composed of elite athletes, heterogeneously composed with the inclusion of people not habitually sporting but occasionally participating in sport, or if people not actively involved in sport were also recruited. The scoring system rated each paper with a score from 0 to 3 for each item. Double-blinded qualitative assessment of the selected papers was independently completed by two authors (NP and FF). Any discrepancies in the assessment of the studies were resolved by further review and agreement of both investigators. The mean of the scores assigned to each item was used to assess the final study quality on a scale ranging between 0 and 3.

## 3. Results

**Figure 1:**
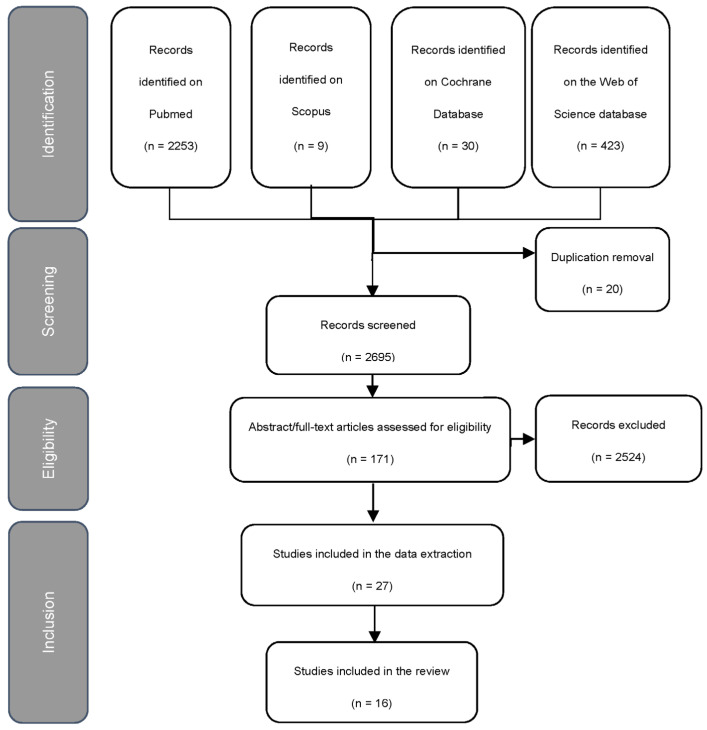
Flowchart of the identification and the selection process for inclusion of articles in the review.

The step-by-step process of identification and application of inclusion and exclusion criteria is represented in the PRISMA flowchart reported in Figure 1.

Out of the 2715 identified unique citations, 2695 articles were assessed after removing duplicates. From this, 2524 titles were excluded, and 171 met the criteria for abstract review. Two researchers carefully evaluated the adherence to the inclusion criteria by examining the abstracts and checking the entire text in case of uncertainty.

In all cases, the researchers reviewed the full text before deciding on eligibility for data extraction.

Twenty-seven studies qualified for data extraction (percentage of agreement of 96.51%; κ: 0.731). Three manuscripts were excluded during the data extraction because they included the same data from previous studies during the same expedition [35,36], and eight were excluded because they did not fit the inclusion criteria for the study. This left 16 papers, for which there was a systematic review.

Telemedicine was used in high-mountain climbing and mountaineering (37.5%; n = 6), skiing and winter sports (18.7%; n = 3), water sports such as windsurfing and swimming in extreme conditions, and diving (25%; n = 4), and long-distance land sports such as endurance running and triathlon (18.7%; n = 3).

The list of the selected papers with the demographic data relative to the studied population, the investigated medical condition/parameter, the assessed parameters, and the limitations of the transmitted data are presented in Table 3. The results of the quality assessment of the studies are reported in Table 4.

Eleven of the selected papers only included fewer than ten participants, and the demographic description of the subjects studied was optimal in only 50% (n = 8) of the studies.

The topic of accuracy was mentioned in 81.2% (n = 13) of the studies; however, only in 50% of the studies (n = 8) was an assessment of the accuracy of telemedicine reported. In three cases, the comparison of the results of telemedicine was made with those obtained from traditional non-telemedical evaluations [46,47,49]; in two cases, the results were evaluated by the doctor involved in the study [35,43]; in one case, the data obtained was examined by a blinded expert [45]; in one case, the results were compared with those of two previously published studies [48]; and in one case, the accuracy as perceived by the athletes involved in the study was evaluated [50].

When a qualitative assessment was reported, telemedicine results were rated as successful to highly successful [35], with no discernible differences compared with the results obtained with a traditional approach [43]. Similarly, regarding sonography, the quality of images was rated as excellent [45].

When a quantitative assessment was obtained, the rate of adequacy ranged between 96.7% and 100% [46,49], while a substantial agreement was found (Cohen’s κ coefficient= 0.721 [47]) when telemedicine was compared with clinical assessment.

Three studies reported that the rate of artefacts ranged between ≤5% to 16.7% [40,46,48], while instrumentation problems that temporarily hampered signal transmission were reported in five studies [35,36,38,41,49].

## 4. Discussion

The articles included in this review show significant heterogeneity in research design and study scope. Some aimed to evaluate specific physiological parameters during sports practice in extreme environments; others assessed the feasibility and effectiveness of using telemedicine in these contexts.

The first aspect worthy of consideration in the comparative analysis of the selected studies was the technologies used for vital signs monitoring and data transfer. The first example was from 1962, when Hanson and Tabakin pioneered using radio transmission of electrocardiograms during competitive racing and jumping skiing events at the Middlebury College Snowbowl, a ski area in Vermont [37]. Some years later, Nagasaka et al. recorded electrocardiograms (ECGs) and heart rates (HRs) via telemetry from mountaineers involved in the Nagoya University expedition to Mt. Aconcagua (Argentina) [38]. Much more recently, with the advancement of technology, Kao et al. (2013) were able to transmit the same data in real time during a continuous ascent of Mount Everest (Nepal) [48].

A quantum leap in the continuity of data collection and transmission has followed the introduction of smart clothing.

Smart clothing allows athletes to perform their sports unencumbered. At the same time, physiological (heart rate and respiration), performance (posture and movement), and environmental (temperature and humidity) data are acquired in real time [51]. These features are especially advantageous in extreme sports, where athletes commonly make rapid decisions and acrobatic movements in unfavourable environments, and any interference could be potentially dangerous.

The MagIC system used by Di Rienzo et al. [46] has textile sensors coupled to a portable electronic board embedded in a vest worn for ECG monitoring and allowed the study of the effects of high-altitude hypoxia on sleep and 24 h performance in 30 climbers [46]. The system functioned correctly in 94.4% of sessions and 86.5% of recordings; comparatively, traditional monitoring by Satava et al. had a loss of transmission rate of up to 44% in certain portions of the trek, due to improper affixing of the leads for heart monitoring [36]. Moreover, 90% of the participants found smart clothing comfortable during sports practice [46].

Motion artefacts, caused by the movement between the textile electrodes integrated into the garment and the skin during the movement of the subject, are the most crucial problems to be addressed in smart garments. However, with the MagIC system, the rate of artefacts dropped to ≤5.0%, a value lower than one-third of the values (16.7%) reported with signal collection by traditional ECG recorder [48]. This shows that the problem of artefacts with smart clothing is relative and can be overcome by technical solutions concerning the garment structure, the level of adherence, the position of the electrodes, and the type of yarns.

On-field monitoring of physiological parameters requires special considerations in extreme environments. For example, conventional wired recording systems can interfere with free body movements. Water sports recording systems that use high-voltage alternating current can expose participants to short circuit and electric shock risks. Waterproof recorders powered by low-voltage batteries can be a solution but do not allow monitoring of signals in real time. Telemetry systems, however, have technical limitations in transmitting when subjects are underwater. To overcome these difficulties, Japanese researchers have experimented with ECG measurement using an amphibious telemetry system [39].

Data transfer technology is another critical aspect for transmitting a continuous medical signal, and using mobile phones is possible when sports are practised in network-covered areas.

However, sequential connectivity to the hundreds of network cells the athlete passes during events on a very extensive track may hinder data transmission. This specific problem was addressed by Spethmann et al. [49] in their study on ECG monitoring during marathons using a system based on data transfer via a mobile phone network. Excellent quality of streaming (ECG streaming duration covered 89% of a Holter registration used for comparison) was obtained, and 100% of the heart rhythm disorders could be detected. With this approach, there were no interruptions, even under the extreme workload caused by thousands of mobile phone customers (e.g., athletes and spectators) attending the event. Only one interruption of the ECG streaming occurred over 3 min of transmission.

This method’s applications are in mass ultraendurance sports competitions, which are becoming increasingly popular and can generate significant injuries, including potentially fatal events [52].

Alternatively, satellite telephones transmitting via the INMARSAT network of geostationary telecommunications satellites have been integrated with existing infrastructures such as Integrated Services Digital Network (ISDN) and the Internet [35,36].

Low-Earth-orbiting satellites (LEOS) have also been proposed as modes of transmission to monitor people in remote areas [35].

This review also highlights some critical issues when designing a telemedicine service for sports in extreme environments.

In particular, the need for adequate time, resources, local contacts, and area knowledge emerged from the study carried out during the Amazon Swim Expedition [44]. To overcome local cultural barriers, diplomatic efforts to obtain the help of local governments, the military, and other organisations with infrastructure and human capacity may be critical in remote and austere regions.

Furthermore, when telemedicine is based only on the symptoms described without the support of instrumental data, medico-legal considerations can lead doctors to classify the pathological conditions in categories of greater seriousness, especially in potentially life-threatening situations such as, for example, diving accidents [47].

The main application of telemedicine to sports practice in extreme environments was teleconsultation. However, a remote consultation is usually held between the patient and the treating physician [53]. An articulated and versatile organisational model was adopted during the Everest Extreme Expeditions (E3) at the Everest Base Camp [35,36].

Teleconsulting was held by an entire medical support team composed of specialists from internal medicine, surgery, and radiology who evaluated and discussed the clinical cases presented by the Base Camp team daily. Thanks to technological support, doctors had appropriate clinical history, viewing of the patients, auscultation with a digital stethoscope, ultrasound images, and laboratory values. Specifically, echographic images were transmitted in real time and allowed evaluation of the thoracic cavity and lungs, facilitating proper treatment, including oxygen and intravenous solutions administration [35,36]. Moreover, the Base Camp team exploited the portable Doppler ultrasound system to quantify blood flow in the carotid, brachial, and posterior tibial arteries of subjects at high altitudes.

In addition to the ultrasound information, pulse oximetry, serum chemistries, and venous blood gas analysis, ECG and noninvasive cardiac output measurements were monitored and recorded, guiding drug administration and monitoring.

Modern medical practice has become highly dependent upon diagnostic imaging to confirm the clinical diagnosis and guide therapies. Ultrasound is the most portable imaging modality and is versatile, safe, dynamic, and relatively cheap. Among the typical applications in sports medicine are ultrasound musculoskeletal examinations.

Sonography is, however, user-dependent, a characteristic that has prompted the development of remote guidance techniques to exploit its potential in wilderness locations [54]. Indeed, the transmission of ultrasound images and remote guidance by expert radiologists can allow the execution of sonographic studies by nonmedical or nontrained personnel [43]. For example, nonphysician personnel with minimal training performed high-fidelity remote-guided ultrasounds on 32 athletes to rule out musculoskeletal sports-related conditions in the upper and lower limbs [43,54].

Besides the musculoskeletal system, sonography can diagnose and follow-up many clinical conditions in extreme environments, including closed abdominal injuries following severe sports trauma [55,56] and examination of arterial flow [35]. Thoracic ultrasound (TUS) [35] can be used in remote locations instead of X-ray examination [35,57], to diagnose and treat potentially life-threatening conditions such as pulmonary oedema in climbers and divers or ultraendurance athletes [45,58,59], as well as haemothorax, haemopericardium, pericardial effusion, pleura and lung injuries, pneumothorax, and consolidations [60]. TUS can also guide interventional procedures such as pericardiocentesis, fluid administration, and chest tube placement [60], thus expanding medical treatment in austere and extreme environments.

Performance monitoring in the continuum to provide immediate first aid in the case of an accident is challenging to apply in extreme sports. On the one hand, in many cases, there is no assistance at all to the practice of these sports; on the other hand, participants may be reluctant to participate in medical projects focused on injury treatment because of their cultural habits [61].

On the contrary, transmitting physiological or biomechanical signals from the sport setting is critical in extreme sports to better understand the specific characteristics of these sports which are influenced by many environment-related variables. This can lead to injury prevention and more effective rehabilitation.

An in-depth understanding of sport-specific patterns is essential to develop performance models for athlete preparation (short-term) and development (long-term) frameworks [62].

In extreme sports, even more than in traditional sports, the noncyclical nature of activities, outdoor settings, and environmental variables hinder data capture, making it challenging to deconstruct the movements in their patterns and collect critical neurophysiological and cinematic data [63].

Sports medicine researchers can use radio-transmitted electrophysiological data captured in the field to comprehensively understand the primary movements involved with different performance techniques, the equipment–athlete interface, and their influence on muscle group recruitment and joint load, namely neuromuscular activation characteristics. This data may shed light on potential pathomechanics that are most likely to result in sudden–acute injuries, such as falls or poorly controlled single-leg landings from jumps during skiing [64], and overuse injuries, aiming at modifying training programs for injury prevention.

For example, Dyson et al. [40] recorded electromyographic (EMG) signals on windsurfers during the beating, reaching, pumping, running, and uphauling activities. Measurements were obtained on up to 13 muscles in 15 s sample periods, and the phasic recruitment assessment was made with an accuracy of 0.01 s.

The measurements identified the characteristic muscle activity patterns in terms of duration, phasing, and magnitude of activity in each muscle during different windsurfing sailing modes and manoeuvres. Measuring EMG on the field allowed the identification and correction of less than-optimal techniques, such as overuse of the trunk muscles rather than arm muscles in pumping or inappropriate order of muscle recruitment. This information may assist in developing training strategies, potentially resulting in performance improvement and injury prevention. In addition, the trainer can use these data to tailor personalised and sport-specific training programs, allowing injury prevention.

Adopting technological advances to reveal athletic gesture characteristics and highlight subtle movement patterns directly in the field can allow adapting the intrinsic dynamics of learners by enhancing their relations with the performance environment and manipulating constraints, leading to individualised movement responses [63].

Finally, physiotherapists can use data captured on the field to create sport-specific retraining programs to be included in the last part of rehabilitation programs, aiming to reproduce the functional positions, movement patterns, and power demands of the practised sport.

For example, Zeglinksi et al. [41] obtained the telemetric electromyography (EMG) of seven trunks and lower extremity muscles in five male elite ski racers involved in a slalom turn in both alpine skiing and inline skating. The results show many similarities in muscle activity patterns between the two sports, allowing the proposal of inline skating as a dry-land training modality for alpine skiing. The results also suggested using inline skating training to more closely mimic the movement patterns of skiing.

Performance monitoring is a particularly relevant approach in complex and noncyclical sporting activities such as windsurfing and skiing. Moreover, outdoor sports involving repetitive athletic movements may benefit from on-field monitoring. For example, the study by Aranki et al. aimed at monitoring and optimising a particular running aspect: cadence [50].

Finally, telemedicine has also been used to design more effective scientific studies to understand thermoregulation in extreme environmental conditions, such as ultraendurance sports practised in hot or humid climates, to prevent heat stroke and update fluid replacement guidelines. For example, Laursen and colleagues used an ingestible telemetric pill [42] to monitor thermoregulation data during a 226 km triathlon race. Measurements were obtained intermittently at even time increments throughout the competition and after the race. Based on the collected data, the authors could update fluid replacement guidelines for triathlon practice. Even more critical for the present study’s purpose, this approach demonstrated the possibility of collecting and telemetrically transmitting an athlete’s core body temperature during sports practice. These data are essential to diagnosing heat-related illnesses, including heat stroke, defined as a core body temperature exceeding 40 °C, which allows physicians and medical teams to recognise this life-threatening emergency promptly.

Among the limitations of this study was the heterogeneity of the existing literature. It examined different sports activities and telemedicine systems used for very different purposes, thus making comparative assessments difficult and quantitative comparisons impossible in most cases.

Second, many studies included small numbers of participants, often fewer than ten, limiting the ability to draw generalisable conclusions.

The quality of the present study may be affected by not using artificial-intelligence-based program for systematic reviews. Despite these limitations, this study allowed us to draw an overall picture of the use of telemedicine in sports practised in extreme environments. Identifying the direction towards which this branch of medicine is expanding can allow researchers to design future studies investigating the issues described, involving more extensive and more homogeneous sample populations.

## 5. Conclusions

Telemedicine is likely to be increasingly involved in sports in extreme environments, both for the possibility of providing specialist advice remotely and supporting the activity of medical and healthcare-allied professionals with physiological and biomechanical data collected in the field.

Smart clothes promise to represent fundamental tools soon, because they respond to the need to use solutions not influenced by the movement of athletes engaged in highly demanding performances.

Among the support systems for diagnosis and medical procedures, ultrasound will increasingly play a crucial role due to its diagnostic power and versatility, thanks to portable devices and the possibility of remote guidance by expert radiologists.

Data transmission systems can be very variable. They can exploit widespread systems, such as mobile phone networks or the Internet, with low-Earth-orbiting satellites representing a possible alternative.

Finally, when organising telemedicine services in austere environments, several critical issues must be considered: the logistical aspects, the cultural ones, and the limitations of telemedicine linked to considerations of a medico-legal nature in the local legal systems.

Therefore, an evolution of legal systems is also necessary to reduce the spread of defensive medicine attitudes when doctors must decide remotely for potentially life-threatening issues.

## Figures and Tables

**Table 1:** Summary of inclusion and exclusion criteria.

Inclusion Criteria			Exclusion Criteria
1. (At Least One among the Following Criteria Must Be Satisfied)	2. The Following Criteria Must Be Satisfied	3. The Following Criteria Must Be Satisfied	
Studies on telemedicine’s efficacy, efficiency, or results and a comparison with traditional medicine. Studies on telemedicine, e-health, or telehealth systems and methods. Studies relating telemedicine use for monitoring performance, vital parameters, health status or consultations, or treatments. Studies about the training of professional and amateur athletes or sportsmen.	Studies relating to all sport disciplines practised outdoors.	Studies relating to healthy participants or participants with pathological conditions resulting from the practice of sport (e.g., injuries or sports illnesses).	Expert opinions Case reports Reviews Non-English-language literature Any paper about the use of physical exercise to prevent pathologies related to a sedentary lifestyle, including technology used to quantify physical activity in sedentary people. Studies on patients affected by illnesses not related to sports practice. Papers related exclusively to sports practised in strictly controlled environments and on a regulated playing fields (e.g., tennis, soccer, and football).

**Table 2:** Criteria adopted for the qualitative assessment of the selected papers. A score ranging between one (*) to three (***) was assigned.

1. Demographic Parameters (Number of Participants, Sex, and Age) Reported	
All parameters reported	***
Two parameters reported	**
One parameter reported	*
Lack of data relative to the Number of Participants, Sex, and Age	-
**2. Representativeness of the exposed cohort**	
Homogeneous cohort of sports participants	***
Heterogeneous cohort, including non-sport people who, for the occasion, took part in a sporting event	**
Heterogeneous groups, including people not involved in sport (bystanders and technical personnel)	*
No description of the derivation of the cohort	-
**3. Quality of the reported data**	
Availability of detailed transmitted data/datasheets from the secure record (e.g., reports)	***
Quantitative resumption of the transmitted data/data from structured surveys	**
Generic qualitative report/self-report	*
No description	-
**4. Assessment of efficiency of the telemedical system**	
Quantitative report by a researcher or qualitative data in a double-blind agreement	***
Semiquantitative report or qualitative data (non-double-blind agreement)	**
Generic description of results	*
No description	-

**Table 3:** Data extracted from the papers included in the review [35,36,37,38,39,40,41,42,43,44,45,46,47,48,49,50].

Authorship	Year of Publication	Sport	Participants			Investigated Medical Condition/Parameter	Applied Technology	Efficacy/Failure Rate
			Number	Males: % (n)	Age: Mean ± DS (Range)			
**Mountain Sports**								
Hanson & Tabakin	1964	Downhill, cross-country, and jumping skiing	4	100% (n = 4)	Unspecified	ECG	Radio-electrocardiography	Quality of ECG: high Artefacts: intermittent, minimal
Nagasaka et al.	1966	Climbing	3	Unspecified	26.6 (22–30)	HR variability, ECG	Radio telemetric system	High signal-noise ratio when the subjects were out of sight from the receiver station
Angood et al.	2000	Climbing	150–250	Unspecified	Unspecified	**Diagnosed medical conditions:** Femur fracture, acute respiratory failure Assessed parameters: heart rate and respiratory rate, body temperature, electrocardiogram, motion, pulse oximetry blood flow in the carotid, brachial, and arteries	Radiologic teleconsultation, portable ultrasound, blood gas analyser, digital spirometry ECG telemetry, surface and core thermometers, blood gas analyser, portable doppler.	Successful to highly successful (Failure rate: 3 conferences)
Kao et al.	2013	Climbing	4	75%; (n = 3)	39 (34–48)	ECG and HR	One lead ECG, HR	Rate of artefact: 16.7% (range: 9.8–30.4)
Zeglinksi et al.	1998	Alpine skiing Inline skating	5	100% (n = 5)	38 ± 4	EMG (right peroneal, anterior tibialis, vastus medialis, biceps femoris, adductors, gluteus maximus, and erector spinae).	Surface Electromyography	Three subjects were required to double the number of tests due to malfunction
Satava et al.	2000	Mountaineering	3	Unspecified	Unspecified	HR, activity level, skin temperature, core temperature	HR monitor, core body thermometer, surface body thermometer (chest and wrist), chest actigraphy (gross body motion), sleep time measurement, geolocation	The vital signs monitoring functioned 95–100% of the time for 2/3 participants
Otto et al.	2009	Mountaineering	2	100% (n = 2)	22 (22–24)	High-altitude pulmonary oedema, pneumothorax	Lung sonography	Quality of the remotely viewed images: good toexcellent (double-anonymised agreement).
Di Rienzo et al.	2010	Mountaineering	30	73.3% (n = 22)	Unspecified	ECG, respiration, movement, altitude sickness.	One ECG lead Piezoresistive plethysmograph (to measure respiratory movements) A three-axis accelerometer	Rate of adequacy: 111/115; 96.7% (artefact rate ≤5.0% ± 0.6%, mean ± SE).
**Ultra-endurance sports**								
Laursen PB, et al.	2006	Triathlon	10	100% (n = 10)	34.7 ± 6	Core temperature (Tcore)	Measurements using ingestible pill telemetry system	Unspecified
Kwon D et al.	2007	Hockey and athletics	32	25% (n = 8)	Unspecified	Groin: sports hernias. Knee: patellar tendon, medial collateral ligament, lateral collateral ligament, medial meniscus Ankle: achilles tendon, anterior and posterior tibiofibular ligaments Shoulder: biceps tendon, supraspinatus tendons, articular cartilage surface, rotator cuff	Remote-guided musculoskeletal ultrasound examinations	No discernible differences between the ultrasound examinations performed at remote locations and those performed in standard conditions.
Spethmann et al.	2014	Endurance running	10	100% (n = 10)	41.7 (35–55).	ECG	Streaming electrocardiogram	ECG streaming duration 3–90% of Holter registration Quality of the Holter sufficientfor analysis: 100% of the athletes Discrepancies inmorphology of arrhythmias ECG streaming vs. Holter: none
Aranki et al.	2018	Long-distance running	6	50% (n = 3)	24.5 (23–26)	Energy expenditure, cadence, speed, heart rate	Remote monitoring of running cadence (steps per minute) through a mobile remote health system (running coach).	Single heart-rate measurements needed to be more accurate
**Watersports**								
Yamaguchi et al.	1993	Underwater swimming	7	100% (n = 7)	29 (22–41)	ECG	Amphibious telemetry	ECG signals obtained were unsatisfactory for detailed classification of arrhythmias
Dyson et al.	1996	Windsurfing	6	50% (n = 3)	Unspecified	EMG activity of 13 muscles ECG	EMG and ECG	In 16% of the participants, the ECG muscle artefact partially corrupted the recording
Latifi et al.	2009	Ultraendurance swimming	Unspecified	Unspecified	Unspecified	Exhaustion, dehydration, sunburn, hypertension, dizziness, wound debridement, infectious tropical disease, sexually transmitted diseases, skin conditions, gastroenteritis, influenza, malarial prophylaxis, prophylaxis for infections, clinical nutrition supplementation, psychotherapy, muscle cramps, arrhythmias, tooth extractions, gynaecological surgery, ankle sprains, allergic food reactions, asthma exacerbation, neurological evaluations, sea sickness, diagnosis of sigmoid volvulus, gastro-esophageal reflux disease	Vital signs monitoring (monitoring of heart rate, blood oxygen saturation, blood pressure) Physical examination Specialist consultations Sonography	Unspecified
Wölfel et al.	2011	Diving	63	79.3 (n = 50)	39.5 (22–70)	Decompression illness (DCI)	Phone calls using a specific protocol	Coherence between hotline assessment and clinical assessment: good (95% confidence interval: 0.551–0.864. Cohen’s κ coefficient = 0.721)

**Table 4:** Results of the quality assessment of the studies. A score ranging between one (*) to three (***) was assigned.

Authors	Demographic Description of the Sample	Representativeness of the Exposed Cohort of Sports Participants	Quality of the Reported Data	Assessment of Efficiency of the Telemedical System	Final Study Quality
Angood et al.	-	*	-	*	0.5
Latifi et al.	-	*	*	-	0.5
Kwon et al.	**	***	*	*	1.75
Satava et al.	*	***	**	***	2.25
Di Rienzo et al.	**	**	**	***	2.25
Otto et al.	***	*	***	***	2.5
Nagasaka et al.	**	**	**	*	1.75
Kao et al.	***	***	***	***	3
Spethmann et al.	***	***	***	***	3
Wölfel et al.	***	**	**	*	2
Dyson et al.	**	***	**	*	2
Laursen et al.	***	***	**	-	2
Zeglinksi et al.	***	***	**	*	2.25
Aranki et al.	***	**	**	*	2
Hanson and Tabakin.	**	***	***	*	2.25
Yamaguchi et al.	***	**	**	*	2

## Data Availability

Data is contained within the article or Supplementary Material.

## Supplementary Materials

The following supporting information can be downloaded at: https://www.mdpi.com/article/10.3390/ijerph20146371/s1.
