# Supplementary material for: Telemedicine in Sports under Extreme Conditions: Data Transmission, Remote Medical Consultations, and Diagnostic Imaging
Source: Int J Environ Res Public Health. 2023 Jul 15;20(14):6371. doi: 10.3390/ijerph20146371 (PMC10380087; doi:10.3390/ijerph20146371)
Supplement: Supplementary file 1 [file ijerph-20-06371-s001.zip › ijerph-2095360-supplementary.pdf]

Supplementary Material 1

Search string launched on PubMed.

((((Tele*[tiab]) OR ("Telemedicine"[mh]) OR ("Teleradiology"[mh]) OR ("Telerehabilitation"[mh]) OR (("Rehabilitation" [mh]) AND (Tele*)) OR ("Telepathology" [mh]) OR ("Telenursing" [mh]) OR ("Remote consultation" [mh]) OR ("Emergency Medical Service Communication Systems" [mh]) OR ("Internet-Based Intervention" [mh])) AND ((Sports [mh]) OR ("Sports Medicine" [mh])))

Search string launched on WOS:

(Telemedicine OR Teleradiology OR Telerehabilitation OR Telepathology OR Telenursing OR Remote Consultation) AND (Sports OR Sports Medicine)
ALL fields
